# Seasonal Variation in Selected Biochemical Traits in the Leaves of Co-Occurring Invasive and Native Plant Species under Mediterranean Conditions

**DOI:** 10.3390/plants11091171

**Published:** 2022-04-26

**Authors:** Maria Cristina Morais, João Alexandre Cabral, Berta Gonçalves

**Affiliations:** Centre for the Research and Technology of Agro-Environmental and Biological Sciences (CITAB), Institute for Innovation, Capacity Building and Sustainability of Agri-Food Production (Inov4Agro), University of Trás-os-Montes e Alto Douro (UTAD), Quinta de Prados, 5001-801 Vila Real, Portugal; jcabral@utad.pt (J.A.C.); bertag@utad.pt (B.G.)

**Keywords:** plant-functional traits, photosynthetic pigments, non-structural carbohydrates, phenolic compounds, lipid peroxidation, *Hakea sericea*, *Acacia melanoxylon*, *Pinus pinaster*

## Abstract

The success of invasive alien species (IAS) is often linked to differences in functional traits in relation to other, either native or non-invasive, species. Two of the most problematic IAS in the Mediterranean area belong to *Hakea* and *Acacia* genera that often invade pine plantations. Therefore, the aim of this study was to assess the seasonal variations in photosynthetic pigments, total phenolics, and non-structural carbohydrates (NSC), including total soluble sugars (SS) and starch (St), and lipid peroxidation, in terms of malondialdehyde (MDA) in the leaves of evergreen species, two IAS (*Hakea sericea* and *Acacia melanoxylon*) and one native (*Pinus pinaster*), throughout 2019. All parameters showed a pronounced seasonal variability while also differing across species. Generally, the lowest contents of photosynthetic pigments, phenolics and SS were noted in early spring, along with the highest St and NSC values. On the other hand, higher photosynthetic pigment and lower NSC contents were measured in early autumn and early winter. When these parameters were compared across the three species, the IAS had significantly higher content of photosynthetic pigments, mainly chlorophyll *b* and total chlorophyll, and lower total phenolics and MDA concentrations in their leaves than *Pinus pinaster*. Differences in seasonal patterns were also observed. *Hakea sericea* and *Acacia melanoxylon* had considerably higher chlorophyll, SS and NSC contents in the early autumn, while *Pinus pinaster* had higher St and MDA contents in early summer. Overall, the biochemical characteristics of leaves of the studied IAS can explain their success in the Mediterranean area, in terms of tolerance to stressful environmental conditions.

## 1. Introduction

Biological invasion by non-native plant species is widely recognized as a serious threat to biodiversity. They disrupt the ecosystem integrity by displacing desirable native/endemic communities [1], altering the functioning of the ecosystem [2], impacting human health [3] and well-being [4], and causing massive economic losses [5]. The great ability of invasive alien species (IAS) to establish and spread in a new habitat, thereby altering the ecosystem integrity, is associated with several functional traits, such as short life cycle and early maturity, higher reproductive, growth and dispersal rates [6], and high plasticity [7], which make them better competitors than native species and maximize their invasion potential.

In Portugal, serious ecological and economic problems caused by IAS have been recognized by law since 1999 [8]. Among invasive taxa, *Acacia* and *Hakea* are two of the most problematic genera established in the national territory, with *Acacia melanoxylon* R.Br. (Fabaceae) and *Hakea sericea* Schrad. and J. C. Wendl. (Proteaceae) being considered aggressive IAS [9]. *Hakea sericea* is an evergreen woody species, originally from south-eastern Australia [10], characterized by very thorny leaves and woody follicles that contain two winged seeds [11]. *Acacia melanoxylon*, a perennial tree, native to southeast Australia and Tasmania, and akin to other *Acacia* species, is a very prolific seed producer and characterized by vigorous resprouting from stump or root suckers [12]. This IAS also synthetizes allelopathic compounds that inhibit the growth of other plants [13,14], favoring its prevalence in the invaded area. In Portugal, both IAS invade a wide range of habitats, including natural habitats, disturbed areas (e.g., road margins), forest margins, and areas associated with *Pinus pinaster* forests [9]. Their prevalence and spread in the Mediterranean area are connected to fire. In the case of *Hakea sericea*, fire stimulates seed release from woody follicles [11], while for both species, fire stimulates germination and the establishment of dense thickets that can suppress native communities [9].

Available evidence indicates that the great ability of IAS to occur at high densities could be ascribed to their superior photosynthetic capacity [15,16] and carbohydrate partitioning [16,17]. For example, Oliveira et al. [18] suggested that the higher chlorophyll/carotenoids and chlorophyll *a/b* ratios, and leaf protein contents in the IAS *Prosopis juliflora*, relative to the native *Anadenanthera colubrina,* can explain its invasive potential. According to Zheng et al. [19], increased chlorophyll *a*/*b* ratio, may be beneficial for the survival of *Mikania micrantha*, a perennial herbaceous creeping vine, under harsh conditions. Similarly, Li et al. [17] argued that higher soluble sugars and lower starch content in the leaves of the invasive Chinese tallow tree (*Triadica sebifera*) is an efficient strategy for increasing metabolic energy, which results in faster growth relative to the native species. Higher accumulation of soluble sugars and starch, commonly known as non-structural carbohydrates (NSC), is frequently associated with the better physiological performance of plants, especially in periods characterized by water shortage and high temperature, typical of summer months in the Mediterranean climate [20]. In this context, Souza et al. [16] demonstrated that the invasive grass *Melinis minutiflora* possesses higher NSC content in periods when the photosynthetic rate is reduced, when compared to the native *Echinolaena inflexa*, which conferred physiological advantages. This ability of an IAS to adapt to variable conditions (phenotypic plasticity) can improve its chances for further expansion [21], enhancing its invasion success [22].

The photosynthetic pigments and NSC contents are highly variable and generally exhibit seasonal fluctuations [16,23,24,25,26,27,28]. Such variability can also occur between species, life habits, plant tissues and biomes [29], and in response to environmental conditions, such as light, temperature, and water availability [30,31]. As these environmental changes can also act as stressors leading to oxidative damage [32], malondialdehyde (MDA) is recognized as one of the most reliable markers of oxidative stress [33]. In addition, phenolic compounds, acting as non-enzymatic antioxidant metabolites, play a key role in the protection against environmental stress conditions [34]. Thus, a better knowledge of the leaf biochemical traits of IAS and how they differ from those of native species is of great importance for understanding the invasion mechanisms, but such studies are presently lacking. This gap in extant knowledge has motivated the presented study, as a part of which, variations in the biochemical traits across seasons and species were examined, focusing on two IAS (*Acacia melanoxylon* and *Hakea sericea*) and one native (*Pinus pinaster*) species, commonly found in Mediterranean areas. Specifically, the contents of photosynthetic pigments (chlorophyll *a* and *b*, total chlorophyll and carotenoids), non-structural carbohydrates (including soluble sugars and starch), and malondialdehyde (MDA), as indicators of oxidative stress and total phenolics, were quantified in all species over four consecutive periods of 2019.

## 2. Materials and Methods

### 2.1. Study Site and Climatic Conditions

The sampling site was located in the Alvão/Marão cordillera, north of Portugal (41°21′ N, 7°49′ W), which integrates the Natura 2000 network. This Site of Community Importance (SCI) covers an area of approximately 58.788 ha. It is characterized by a diverse range of habitats, in which oaks, mainly *Quercus robur* and *Quercus pyrenaica*, and ericaceous and gorse shrubs are the most dominant species. This SCI is also home to other woody species that were planted, such as *Pinus pinaster*, *Pinus sylvestris*, and *Eucalyptus globulus*. However, several patches of invasive alien species (IAS) are also observed, including diverse Acacia species (*Acacia dealbata*, *Acacia longifolia*, and *Acacia melanoxylon*) and *Hakea sericea*. *Hakea sericea* and *Acacia melanoxylon* were introduced in the area at the beginning of the 20th century and their expansion has been associated with the increased occurrence of wildfires. Presently, both IAS occupy different altitudinal zones (400–800 m a.s.l.), replacing native plant species, with a clear dominance of *Hakea sericea*.

**Figure 1:**
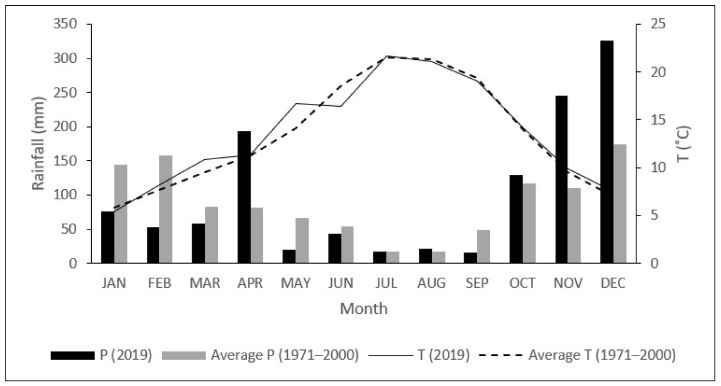
Average monthly Temperature (T, °C) and rainfall (P, mm) in 2019 and 1971–2000.

The SCI Alvão/Marão is an intermediate zone between the Atlantic and Mediterranean climate, with a mean annual rainfall of 1.074 mm, mostly during winter (Figure 1). The average monthly temperature ranges from 5.8 °C in January to 21.5 °C in July, with a mean minimum of −6.5 °C in January, and a mean maximum of 39.8 °C in August (Figure 1), according to the data provided by IPMA (Portuguese Institute for Sea and Atmosphere), for the 1971–2000 period, as measured by the Vila Real Meteorological Station located at 41°18′ N, 7°44′ W (IPMA 2021).

### 2.2. Sampling Site and Data Collection

The sampling site is located in the south-western part of the SCI (41.17° N, 7.59° W), at 450 m a.s.l., where all analyzed species co-occur. Six young plants (about 4 years old) of all species were marked and visited during 2019. Meteorological data (air temperature (°C) and rainfall (mm)) for 2019 were recorded by an automatic meteorological station close to the study area (Figure 1). In 2019, the average monthly temperature was 13.5 °C, with the summer maximum (38.8 °C) in July and the winter minimum (−3.4 °C) in January. The annual precipitation was 1203.6 mm, with about 58% of rainfall recorded between October and December. The lowest and highest rainfall occurred in July (18 mm) and December (326.4 mm), respectively (Figure 1).

Leaf samples were collected at four occasions during 2019: 4 January, DOY (day of the year) 4, early winter; 26 March, DOY 85, early spring; 26 June, DOY 177, early summer, and 2 October, DOY 275, early autumn). On each sampling date, fully insolated 1-year-old leaves at the mid-crown level were collected. To avoid irradiance effects and to ensure consistency, all leaf collections were made between 12:00 and 14:00 h. The samples were placed in plastic bags and were transported to the laboratory under refrigerated conditions. Upon arrival, all samples were immediately frozen in liquid nitrogen and stored at −80 °C for further analysis. They were subsequently lyophilized for one week at −55 °C and converted to a dried powder by grinding.

### 2.3. Biochemical Determinations

#### 2.3.1. Photosynthetic Pigments

The quantification of chlorophylls (chlorophyll *a* (chl*a*), chlorophyll *b* (chl*b*), total chlorophyll (chl*a*+*b*) and total carotenoids (carot)) were carried out on lyophilized leaf samples using the methodology described by Sesták et al. [35] and Lichtenthaler [36], respectively. Both determinations were performed using 80% cold acetone with distilled water (*v*/*v*) and under dim light to avoid photosynthetic pigment degradation.

#### 2.3.2. Total Soluble Sugars and Starch Contents

Total soluble sugars (SS) were extracted by heating the samples in 80% ethanol for 1 h at 80 °C, and the methodology described by Irigoyen et al. [37] was used for quantification. Starch (St) was extracted from the same solid fraction by heating the samples in 30% perchloric acid for 1 h at 60 °C, according to Osaki et al. [38]. Both SS and St were determined by the anthrone method, using glucose as a standard.

#### 2.3.3. Total Phenolics Content

For determining the total phenolics content, the method of Singleton and Rossi [39] was applied using the same extracts as those employed for photosynthetic pigments. The extracts (20 μL) were mixed with 100 μL Folin-Ciocalteu reagent and 80 μL sodium carbonate solution (7.5% *w*/*v*), and then incubated at room temperature for 30 min, after which the absorbance was read at λ = 765 nm with gallic acid serving as the standard.

#### 2.3.4. Lipid Peroxidation

Lipid peroxidation was evaluated by measuring malondialdehyde (MDA) content, following the procedure described by Heat and Parker [40]. Briefly, 10–20 mg of lyophilized leaves was homogenized using 1.0 mL of 0.1% trichloroacetic acid (TCA), before being centrifuged at 15,000 rpm for 15 min at 4 °C. In a test tube (2.0 mL), the reaction mixture containing 1000 μL 0.5% thiobarbituric acid (TBA) diluted in 20% TCA, and 250 μL extract was incubated in a boiling water bath for 30 min. Then, the test tubes were cooled and centrifuged, and the absorbance was read at 532 and 600 nm. MDA content was calculated using the MDA extinction coefficient of 155 mM cm^−1^ and the results were expressed as nmol.mg^−1^ Dry Weight (DW).

All biochemical analyses were conducted using a SPECTROstar Nano Microplate Reader (BMG LABTECH, Ottenberg, Germany).

### 2.4. Statistical Analysis

A repeated-measures analysis of variance (ANOVA) was performed with DOY as the within-subject factor, and species as the between-subject factor. If significant differences related to DOY or species (*p* < 0.05) were noted, the Tukey–Kramer honestly significant differences (HSD) test with the Bonferroni adjustment was performed. The results were expressed as mean ± standard error (SE), *n* = 6. All analyses were conducted in IBM SPSS version 23 for Windows (Orchard Road-Armonk, Armonk, NY, USA).

## 3. Results

### 3.1. Leaf Photosynthetic Pigments

The results reported in Table 1 reveal a significant seasonal variation (*p* < 0.001) in chlorophyll *a* (chl*a*), chlorophyll *b* (chl*b*), total chlorophyll (chl*a*+*b*) and carotenoid (carrot) concentrations. Further examination of the photosynthetic pigment content, with respect to the collection date, revealed that each parameter had the lowest value on DOY 85, while it was relatively constant on the remaining three collection dates. A significant variation was also detected between species (*p* < 0.001). Overall, chl*a*, chl*b* and chl*a*+*b* contents were higher in the IAS, relative to the native species, whereas carrot content was the lowest in the invasive *Acacia melanoxylon*.

**Figure 2:**
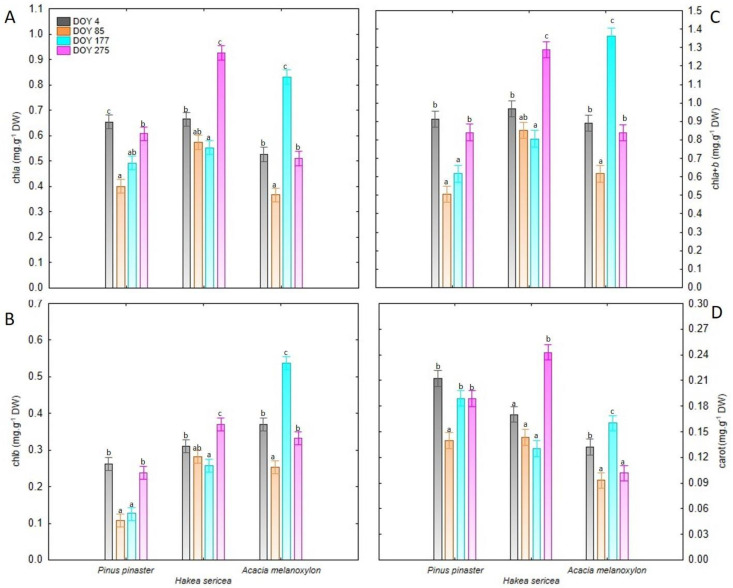
(**A**) Chlorophyll *a* (chl*a*), (**B**) chlorophyll *b* (chl*b*), (**C**) total chlorophyll (chl*a*+*b*), and (**D**) carotenoid (carrot) contents in leaves of all analyzed species (*Pinus pinaster*, *Hakea sericea* and *Acacia melanoxylon*) on the four sampling dates. For each species, different lowercase letters indicate significant differences between day of the year (DOY) according to the Tukey’s HSD test results.

All studied species exhibited distinct seasonal patterns, as indicated by significant DOY × Species interaction (*p* < 0.001). For the native species, the highest chl *a*, chl *b* and chl*a*+*b* values were recorded on DOY 4 and DOY 275, whereas low pigment content (about 50% of the values observed on other dates) was noted on DOY 85 and DOY 177 (Figure 2). On the other hand, *Hakea sericea* presented the highest chl*a*, chl*b*, chl*a*+*b* and carrot contents on DOY 275, but these values were not statistically different from those determined on DOY 4 (Figure 2). Finally, for *Acacia melanoxylon*, the highest photosynthetic pigment concentrations were observed on DOY 177 (Figure 2).

**Figure 3:**
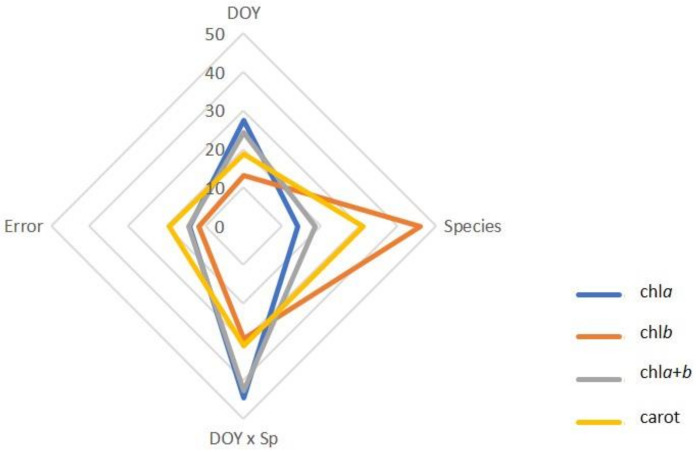
Proportion of the total variance in chlorophyll *a* (chl*a*), chlorophyll *b* (chl*b*), total chlorophyll (chl*a*+*b*), and carotenoids (carrot) by sources of variation (DOY, Species, DOY × Sp, and Error).

Based on the results of the two-way repeated measures ANOVA, Species and the DOY × Species interaction jointly explained 58.6% to 75.0% of the observed variation in photosynthetic pigments (Figure 3).

### 3.2. Non-Structural Carbohydrates

Total soluble sugars (SS), starch (St), and their overall content (NSC) varied significantly throughout the year (Table 2) and did not follow a consistent pattern. For the SS content, the highest value was noted on DOY 4 (88.80 mg g^−1^ DW), after which it decreased by about 23%, reaching an average minimum of 68.54 mg g^−1^ that persisted for the rest of the year. On the other hand, St content was the lowest on DOY 4 (63.13 mg g^−1^ DW), after which it increased, reaching a maximum on DOY 85 (117.08 mg g^−1^ DW). In turn, St content determined on DOY 177 and DOY 275 was similar, but higher than that observed on DOY 4 and lower than that observed on DOY 85. The NSC content reached a maximum on DOY 85 (184.39 mg g^−1^ DW), while it was relatively constant (about 153.00 mg g^−1^ DW) across all other measurement dates. The SS/St ratio also exhibited a seasonal trend, being significantly higher on DOY 4 (1.38), followed by DOY 177 (0.95), DOY 275 (0.83), and finally, DOY 85 (0.57).

**Figure 4:**
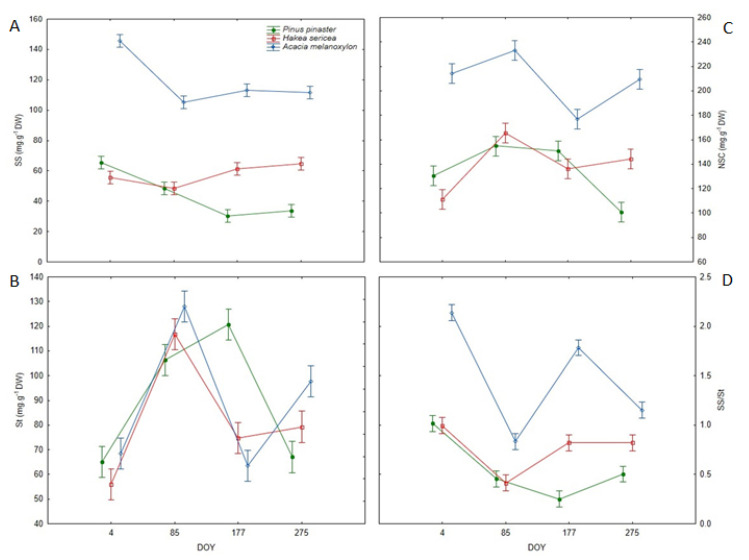
(**A**) Soluble sugars (SS), (**B**) starch (St), and (**C**) non-structural carbohydrate (NSC) content, and the (**D**) soluble sugars/starch ratio (SS/St) in the leaves of all analyzed species (*Pinus pinaster*, *Hakea sericea* and *Acacia melanoxylon*) on the four sampling dates.

The SS, St and NSC contents, as well as the SS to St ratio (SS/St), varied significantly among species (Table 2). The invasive tree *Acacia melanoxylon* had 2.1- and 2.7-times higher SS content than *Hakea sericea* and *Pinus pinaster*, respectively. The St content in *Hakea sericea* was significantly lower than that observed in *Pinus pinaster* and *Acacia melanoxylon*. In turn, NSC contents in *Acacia melanoxylon* (208.29 mg g^−1^ DW) differed substantially from the values determined for the other species (136.73 mg g^−1^ DW). Regarding the SS/St, the highest ratio (1.48) was noted for *Acacia melanoxylon,* followed by *Hakea sericea* (0.76) and *Pinus pinaster* (0.56). *Acacia melanoxylon* was also characterized by the highest contribution of SS to NSC pool, while St was the dominant form in *Hakea sericea* and *Pinus pinaster* (Figure 4).

Analyses further revealed temporal variations among the examined species, as indicated by the significant DOY × Species interactions (Table 2, Figure 4). Overall, SS content in *Pinus pinaster* and *Acacia melanoxylon* was the highest on DOY 4 and decreased thereafter, whereas in *Hakea sericea,* the highest SS content was determined on DOY 177 and DOY 275 (Figure 4). The St content in the native tree exhibited an upward trend from DOY 4 to DOY 177 and declined by about 50% on DOY 275, reaching a similar value to that measured on DOY 4. In turn, St content in *Hakea sericea* increased from DOY 4 to DOY 85, after which it declined by about 36%, as measured on DOY 177 and DOY 275. In *Acacia melanoxylon*, following a 1.9-fold increase from DOY 4 to DOY 85, St content decreased by about 50% on DOY 177 and increased again on DOY 275 to 97.73 mg g^−1^ DW. The NSC content in both IAS showed a trend that mirrored the pattern observed for St. In *Pinus pinaster*, NSC content increased from DOY 4 to DOY 177, and was significantly lower on DOY 275 (Figure 4). The mean SS/St ratio was consistently higher in *Acacia melanoxylon* compared to the other species (Figure 4). Both IAS presented the highest ratio on DOY 4 and the lowest on DOY 85. In the native species, a significant variation in the SS/St was noted, with the highest value (DOY 4) 2.4-times greater than the lowest value (DOY 177).

**Figure 5:**
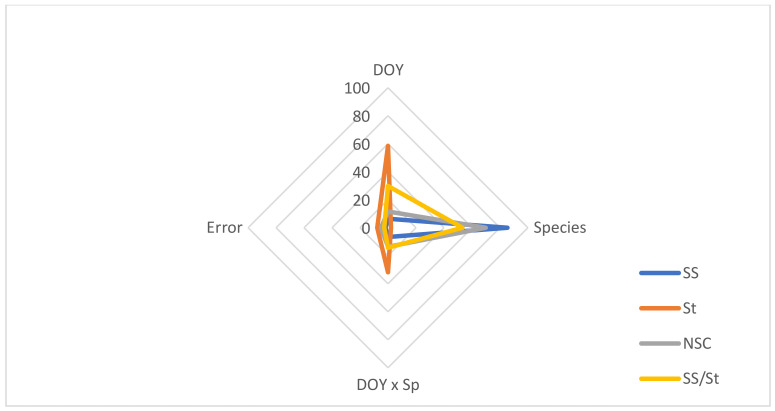
Proportion of the total variance in soluble sugars (SS), starch (St), non-structural carbohydrates (NSC), and soluble sugars/starch ratio (SS/St) by sources of variation (DOY, Species, DOY × Sp, and Error).

The decomposition of variance by sources of variation indicated that Species explained 69.8–85.3% of the variation found in SS, NSC and SS/St, while DOY was the major source of variation in St, explaining about 59% (Figure 5).

### 3.3. Total Phenolic and MDA Contents

When the total phenolics content was analyzed, a significant effect of DOY (31.5%, *p* < 0.001, *F* = 38.743), Species (17.8%, *p* < 0.001, *F* = 25.140) and DOY × Species (38.4%, *p* < 0.001, *F* = 23.555) was observed. The highest values of phenolics were observed on DOY 4 and DOY 177, and the lowest values on DOY 85. The phenolic content in the native species was 21.8% and 17.8% higher than those determined in the IAS *Hakea sericea* and *Acacia melanoxylon*, respectively. In all species, the total phenolics content was lower on DOY 85 and reached the highest value on DOY 4 for *Pinus pinaster* and *Acacia melanoxylon*, and on DOY 177 for *Hakea sericea* (Table 3).

DOY (6.9%, *p* < 0.001, *F* = 82.601), Species (69.6%, *p* < 0.001, *F* = 1737.614) and DOY × Species (21.9%, *p* < 0.001, *F* = 131.460) were also significant sources of variation in the MDA content. Specifically, lower MDA concentrations were noted on DOY 85 and DOY 275 (0.230 nmol mg^−1^ DW and 0.231 nmol mg^−1^ DW, respectively) when compared with DOY 4 and DOY 177. The greatest MDA content was measured for *Pinus pinaster* (0.466 nmol mg^−1^), followed by *Acacia melanoxylon* (0.302 nmol mg^−1^) and *Hakea sericea* (0.076 nmol mg^−1^). Different species-specific patterns were also observed across the year. In *Pinus pinaster* and *Hakea sericea,* MDA tended to peak on DOY 4 and DOY 177, whereas *Acacia melanoxylon* reached the highest MDA content on DOY 85 and DOY 275 (Table 3).

## 4. Discussion

Mediterranean ecosystems are known to experience strong seasonal variability in precipitation and temperature, with profound implications for plant physiological performance [41]. In particular, the environment can induce changes in leaf biochemical traits (e.g., photosynthetic pigments, soluble carbohydrates, and secondary metabolites) that affect plant response and adaptation to variable conditions [31]. In the current study, all considered biochemical leaf traits were significantly affected by season, species and their interaction, and this is consistent with the findings yielded by several studies involving Mediterranean plants [25,32].

The photosynthetic pigment (chlorophyll *a*, chlorophyll *b*, total chlorophyll and carotenoids) contents were highly variable throughout the year, with the lowest values recorded in early spring (DOY 85), after which they remained relatively constant. The reduction in chlorophyll and carotenoid contents detected at the beginning of spring were also reported for other Mediterranean evergreen species [42] and could be associated with the short photoperiod and low temperatures typical of Mediterranean winters, which were observed in the study area during 2019. As expected, elevated temperatures and longer photoperiods led to a general recovery in the leaf chlorophyll and carotenoid concentrations, which increased considerably and remained at that level in summer and autumn. Similar findings were reported for *Pinus nigra* subsp. *pallasiana* [43], *Pinus canariensis* [44], and *Dendrocalamus strictus* [45]. Although the occurrence of severe stress conditions in summer generally hinders leaf pigment production [42], this adverse effect was not observed in the current study. The accumulation of antioxidants, such as carotenoids, during summer and autumn seems to be a protective response against oxidative damage caused by an excess of absorbed radiation [44,46] under such conditions. Moreover, during summer, phenolic contents increased in the analyzed species. The accumulation of these secondary metabolites can be viewed as a defense mechanism that helps in scavenging reactive oxygen species [47], thus, avoiding oxidative stress induced by summer stress.

Leaf carbohydrate contents also exhibited seasonal (albeit different) trends in non-structural carbohydrates (NSC) and their soluble sugars and starch components. Substantial variation in the levels of carbohydrate reserves throughout the year have been reported for several deciduous and evergreen species in the Mediterranean region, in response to varying environmental conditions [28,48,49]. According to our results, soluble sugars content was much higher in early winter than in other seasons, while the opposite trend was noted for starch content, indicating an inter-conversion between the two carbohydrate components [48]. The higher accumulation of soluble sugars at the beginning of the year, confirmed by the increased ratio of sugars to starch, may contribute to plant acclimation to less favorable conditions during winter that are typical of the Mediterranean climate. Cavender-Bares et al. [50] and Ugarte et al. [51] highlighted the important role of sugar accumulation in the enhancement of cold tolerance of several Mediterranean plants, allowing them to maintain their activity at lower temperatures. After winter, the soluble sugars content in the species analyzed in this study decreased by about 23%, and this decline was accompanied by a decrease in soluble sugars to starch ratio, indicating that a greater proportion of available carbohydrates was allocated to storage [52]. However, this pattern appears to be species-specific, since about 85% of the variation in soluble sugars was explained by species, possibly reflecting differences in their phenological cycles [17,28,29,52,53]. Indeed, the three species at the focus of this investigation reach maturity at different ages. The native *Pinus pinaster* and the invasive *Acacia melanoxylon* did not start flowering and fruiting during the study period, while the first flowers were noted on the other invasive species, *Hakea sericea*, in January; the first green (immature) fruits emerged in early spring and their maturation was evident in early summer. Despite the phenological differences across species, the invasive species contained more soluble sugars than the native, especially in summer, giving them an advantage when subjected to more stressful conditions. In contrast to soluble sugars, most of the variation in starch content (about 60%) was related to the sampling date, followed by DOY × Species interaction (about 30%). These findings are in line with other seasonal studies under Mediterranean conditions. Davidson et al. [28] noted that starch content displayed significant seasonality with species-specific differences. In both invasive species examined in this study, the starch content peaked in early spring, decreased to a minimum in early summer, and increased again in early autumn. In the native species, starch concentrations were the highest in early summer, after which they declined and remained low during autumn and early winter. The elevation of starch content from spring to early summer coincides with more favorable climatic conditions for photosynthetic activity in the Mediterranean and its subsequent decrease reflects the carbohydrates conversion from starch to soluble sugars [52]. The invasive species exhibited similar seasonal NSC dynamics, while a distinct pattern was noted for the native species, especially in summer, suggesting a lower defense against summer stress [28].

Photosynthetic pigment levels also differed across the studied species, which explained 14–46% of the total variation. *Hakea sericea* and *Acacia melanoxylon* had a significantly (1.04- to 2.03-times) higher chlorophyll content compared to the native species, *Pinus pinaster*, indicating their greater carbon-capture capacity [15]. It has been suggested that photosynthetic traits related to carbon could constitute an advantage in terms of growth and productivity, which would facilitate the spread and success of invasive plants [15,26]. Significant season × species interactions were found for leaf pigments, reflecting different seasonal variation across analyzed species. In *Pinus pinaster*, leaf pigments reached the highest content in the autumn–winter period, while their values peaked in summer in both invasive species, denoting different strategies for resource acquisition and utilization [54]. Apart from chlorophylls, carotenoids are also considered a good indicator for plant response to stressful conditions [46]. The higher total content of carotenoids observed in the native species, especially at early winter, may, thus, suggest that this secondary metabolite can be an important mechanism for alleviating stress caused by lower temperatures [55]. On the other hand, greater accumulation of carotenoids and phenolics in leaves of both invasive species in summer, may help mitigate the negative influence of summer stress in these species. These observations are in line with those reported by in Zunzunegui et al. [56] for *Oenothera drummondii*. In addition, the low accumulation of end products of lipid peroxidation (proxied by MDA content), observed in leaves of both invasive species, in relation to the native one, indicates their capacity to tolerate oxidative stress under adverse environmental conditions. Altogether, these can be interpreted as “intelligent” behavior [57].

In conclusion, the results presented here indicate significant seasonal fluctuations in the measured parameters, as well as marked differences between the native and the invasive species. The superior performance of both IAS in comparison to the native tree suggests that these species possess leaf biochemical traits that allow them to better acclimate to stressful climatic conditions, which may explain their success and prevalence in the Mediterranean. Therefore, hotter and warmer environmental conditions could potentially favor these IAS by enhancing their competitiveness and their invasive ability.

## Figures and Tables

**Table 1:** Concentration of photosynthetic pigments (chl*a*, chl*b*, chl*a*+*b*, and carrot) from leaves of *Pinus pinaster*, *Hakea sericea* and *Acacia melanoxylon* during 2019. Following two-way repeated measures ANOVA, for each trait, different lowercase letters indicate significant differences (*p* < 0.05) among day of the year (DOY) and different capital letters represent significant differences among species according to the Tukey’s HSD test results. *** *p* < 0.001.

**Source of Variation**		**chl*a*** **(mg.g^−1^ DW)**	**chl*b*** **(mg.g^−1^ DW)**	**chl*a*+*b*** **(mg.g^−1^ DW)**	**carot** **(mg.g^−1^ DW)**
**Time**	DOY 4	0.615 ± 0.024 b	0.314 ± 0.017 b	0.924 ± 0.032 b	0.171 ± 0.009 b
	DOY 85	0.447 ± 0.026 a	0.214 ± 0.020 a	0.658 ± 0.041 a	0.125 ± 0.007 a
	DOY 177	0.625 ± 0.037 bc	0.307 ± 0.042 b	0.928 ± 0.079 b	0.160 ± 0.008 b
	DOY 275	0.681 ± 0.045 c	0.313 ± 0.016 b	0.990 ± 0.056 b	0.178 ± 0.015 b
**Species**	*Pinus pinaster*	0.539 ± 0.026 A	0.183 ± 0.018 A	0.719 ± 0.043 A	0.182 ± 0.007 B
	*Hakea sericea*	0.680 ± 0.033 B	0.305 ± 0.010 B	0.979 ± 0.043 B	0.172 ± 0.010 B
	*Acacia melanoxylon*	0.559 ± 0.036 A	0.373 ± 0.023 C	0.927 ± 0.059 B	0.122 ± 0.006 A
**Two-way Repeated measures ANOVA**					
	DOY	***	***	***	***
	Species (Sp)	***	***	***	***
	DOY × Sp	***	***	***	***

**Table 2:** Results of two-way repeated measures ANOVA (*F* and *p*-values) on the effect of day of year (DOY), Species (Sp) and interaction between DOY and Species (DOY x Sp) on the total soluble sugars (SS), starch (St), non-structural carbohydrates (NSC), and SS/St ratio. *** *p* < 0.001.

Source of Variation		SS		St		NSC		SS/St	
	d.f.	*F*	*p*	*F*	*p*	*F*	*p*	*F*	*p*
DOY	3	71.937	***	129.692	***	41.940	***	203.702	***
Species (Sp)	2	1454.332	***	18.372	***	677.127	***	702.720	***
DOY × Sp	6	37.566	***	35.260	***	24.655	***	49.402	***

**Table 3:** Total phenolic and MDA contents in the leaves of all analyzed species (*Pinus pinaster*, *Hakea sericea* and *Acacia melanoxylon*) on the four sampling dates (DOY 4, DOY 85, DOY 177 and DOY 275). Within each row, different lowercase letters indicate significant differences (*p* < 0.05) between species according to Tukey’s HSD test results.

Biochemical Trait	DOY	Species		
		*Pinus pinaster*	*Hakea sericea*	*Acacia melanoxylon*
Total phenolics (mg g^−1^ DW)	4	47.848 ± 2.756 b	27.719 ± 0.944 a	32.161 ± 0.311 a
	85	23.523 ± 1.691 ab	20.745 ± 0.860 a	27.672 ± 0.889 b
	177	40.447 ± 0.808 b	35.965 ± 2.410 b	30.209 ± 0.711 a
	275	32.115 ± 1.059 a	32.778 ± 0.932 a	32.124 ± 0.726 a
MDA (nmol.mg^−1^ DW)	4	0.605 ± 0.007 c	0.115 ± 0.002 a	0.264 ± 0.009 b
	85	0.272 ± 0.019 b	0.045 ± 0.001 a	0.376 ± 0.003 c
	177	0.663 ± 0.026 c	0.085 ± 0.002 a	0.258 ± 0.005 b
	275	0.326 ± 0.011 b	0.058 ± 0.001 a	0.310 ± 0.003 b

## Data Availability

Not applicable.
